# Subjective Cognitive Decline Is More Accurate When Metamemory Is Better

**DOI:** 10.3389/fnagi.2022.787552

**Published:** 2022-03-09

**Authors:** Silvia Chapman, Jillian L. Joyce, Megan S. Barker, Preeti Sunderaraman, Sandra Rizer, Edward D. Huey, Jordan Dworkin, Yian Gu, Stephanie Cosentino

**Affiliations:** ^1^Cognitive Neuroscience Division, Columbia University Irving Medical Center, New York, NY, United States; ^2^Taub Institute for Research on Alzheimer’s Disease and the Aging Brain, Columbia University Irving Medical Center, New York, NY, United States; ^3^Gertrude H. Sergievsky Center, Columbia University Irving Medical Center, New York, NY, United States; ^4^Department of Neurology, Columbia University Irving Medical Center, New York, NY, United States; ^5^Department of Neurology, Boston University School of Medicine, Boston, MA, United States; ^6^Brain Aging Program, Framingham Heart Study, Framingham, MA, United States; ^7^Department of Psychiatry, Columbia University Irving Medical Center, New York, NY, United States

**Keywords:** subjective cognitive decline, metamemory, preclinical Alzheimer’s disease, self awareness, early cognitive dysfunction

## Abstract

**Objective:**

Subjective cognitive decline (SCD) has emerged as one of the first manifestations of Alzheimer’s disease (AD). However, discrepancies in its relationship with tests of memory and other cognitive abilities have hindered SCD’s diagnostic utility. Inter-individual heterogeneity in metamemory, or memory awareness, and the use of clinical measures of cognition lacking sensitivity to early cognitive dysfunction, may contribute to these discrepancies. We aimed to assess if the relationship between SCD and markers of early cognitive dysfunction is moderated by metamemory abilities.

**Methods:**

The sample included 79 cognitively healthy older adults (77% female, 68% White, and 32% Black participants) with a mean age of 74.4 (*SD* = 6.1) and 15.9 (*SD* = 2.7) years of education. Metamemory was assessed using an episodic Feeling of Knowing test with four 5-item trials. Outcome measures included a resolution metric defined as a gamma correlation reflecting the accuracy of item-level predictions (“Will you know the correct answer?”). Early cognitive dysfunction was measured through the Loewenstein-Acevedo Scale for Semantic Interference and Learning (LASSI-L) and the Short-Term Memory Binding Test (STMB), measures sensitive to preclinical AD. SCD was assessed with a 20-item questionnaire that asked participants to compare themselves to others their age on a 7-point Likert scale. Regression analyses examined whether a potential relation between SCD and early cognitive dysfunction was moderated by metamemory.

**Results:**

Subjective cognitive decline was associated with susceptibility to semantic proactive interference such that greater complaints were associated with increased susceptibility to semantic proactive interference (*b* = −0.30, *p* = 0.003) only. Metamemory moderated the association between SCD and susceptibility to and recovery of semantic proactive interference such that those with more accurate metamemory showed a stronger association between increased complaints and susceptibility to semantic proactive interference (*b* = −0.71, *p* = 0.005; *b* = −0.62, *p* = 0.034). Metamemory, however, did not moderate the association of SCD with retroactive semantic interference nor short term memory binding.

**Discussion:**

The accuracy of an individual’s metamemory, specifically their ability to adjust moment to moment predictions in line with their performance, can influence the extent to which SCD maps onto objective cognition. Such self-referential assessment should be considered when interpreting SCD.

## Introduction

Researchers are mapping the earliest end of the Alzheimer’s disease (AD) continuum to identify patients in a critical window for therapeutic intervention (Dubois et al., 2016). While *in vivo* detection of AD pathologies using biomarkers is central to this process (Sperling et al., 2011), it is not sufficient given the imperfect association between neuropathology and clinical manifestation of disease (Negash et al., 2013). Indeed, at least a third of cognitively normal older adults have evidence of pathological AD on autopsy (Negash et al., 2013) or amyloid imaging (Chételat et al., 2013), and the pathological definition of AD continues to be debated (de la Torre, 2004; Castellani and Smith, 2011; Castellani and Perry, 2014). The ongoing questions and controversies surrounding clinical-pathological correlations in AD (Castellani and Smith, 2011; Castellani and Perry, 2014) emphasize the importance of identifying the earliest *clinical* manifestations of disease. Subjective cognitive decline (SCD), defined as the perception of cognitive decline despite normal performance on traditional neuropsychological testing, is likely to be one such early manifestation of illness with studies increasingly pointing to the potential relevance of SCD as an inexpensive and easily obtainable “pre-clinical” marker of AD (Geerlings et al., 1999; Reisberg et al., 2008; Sperling et al., 2011; Rabin et al., 2017; Jessen et al., 2020).

Research in AD as well as in aging generally supports an association between SCD and objective memory both cross-sectionally and longitudinally, and there is emerging evidence of the association between SCD and AD biomarkers (Gilewski et al., 1990; Hertzog et al., 1990; Pearman and Storandt, 2004; Beaudoin and Desrichard, 2011; Amariglio et al., 2012; Perrotin et al., 2012; Hülür et al., 2014; Snitz et al., 2015; Chen et al., 2019, 2021). However, the utility of SCD as a marker of cognitive functioning and biomarker status appears to vary as a function of multiple factors including task factors (e.g., measurement and operationalization issues) and person factors (e.g., individual characteristics) which together obscure its association with objective markers of disease (Schmidt et al., 2001; Jessen et al., 2010; Tandetnik et al., 2015; Ossenkoppele and Jagust, 2017). For example, the perceptions that memory is worse than others of the same age (i.e., age-anchored SCD) maps on more closely to AD biomarkers than perceptions of memory being bad in general, or worse than before, for example (Perrotin et al., 2012; Tandetnik et al., 2015; Chapman et al., 2021). With regard to person factors, there is recognition that personality and mood are likely important in the conceptualization of SCD; however, other factors remained to be explored (Pearman and Storandt, 2004; Slavin et al., 2010; Merema et al., 2013; Steinberg et al., 2013).

From a self-awareness perspective, SCD may be considered a hyperaware state (hypernosognosia) indicative of early dysfunction not yet detectable, or which does not reach a formal threshold for impairment, on clinical neuropsychological measures. As disease progresses, disordered awareness in the form or lack of awareness of deficits (anosognosia) likely follows SCD in a subset of individuals with mild cognitive impairment; this disordered awareness can be a prognostic indicator of disease progression as well as important clinical outcomes (Starkstein, 2014; Vannini et al., 2017; Munro et al., 2018). Knowledge of one’s own cognitive abilities (e.g., metacognition) has been examined extensively in healthy young and older adults (Nelson, 1990; Price et al., 2010; Hertzog and Dunlosky, 2011; Souchay and Isingrini, 2012; Cauvin et al., 2019; Siegel and Castel, 2019; Gagliardi et al., 2020) and has proven useful in understanding the clinical phenomenon of anosognosia, particularly disordered awareness of memory loss (Cosentino et al., 2007; Galeone et al., 2011; Rosen et al., 2014; DeLozier and Davalos, 2016).

Indeed, several groups have used metamemory testing to measure memory awareness in AD, and this type of assessment may offer a unique vantage point into the accuracy of SCD. As a direct measure of one’s memory awareness, metamemory is a critical person factor that should be considered in the interpretation of SCD. Specifically, individuals who demonstrate good metamemory (i.e., who have good awareness of their actual memory function), may be expected to have a more accurate subjective report of cognitive decline than those who have poor metamemory. Despite its clear relevance for understanding the prognostic relevance of SCD, metamemory has rarely been examined in relation to SCD (Buckley et al., 2016; Vannini et al., 2019; Chi et al., 2020; Gagliardi et al., 2020), perhaps because metamemory as a construct evolved primarily in the field of cognitive psychology and is not a formal component of clinical neuropsychological evaluations (Sunderaraman and Cosentino, 2017; Chapman et al., 2020).

The aim of this paper is to examine the extent to which metamemory moderates the relation between SCD and objective memory. As performance on traditional neuropsychological assessments of memory is by definition “normal” in individuals with SCD, we must utilize more challenging and sensitive neuropsychological tests to more rigorously examine the accuracy of SCD. The current study includes two memory measures shown to be sensitive to SCD as well as to AD biomarkers among clinically normal older adults. As stated above, our hypothesis postulates that those with better metamemory will have more accurate SCD; defined as a stronger association between SCD and objective memory testing on sensitive tasks.

## Materials and Methods

### Participants

Participants included in this study were selected from a larger cohort that comprises 157 participants recruited from the Columbia University Medical Center Aging and Dementia Neurology Clinic (*n* = 12) and ongoing aging studies at Taub Institute at Columbia University (*n* = 145). Two clinical cases were referred to the neurology clinic through a memory-concern screener administered in the Columbia University Department of Obstetrics and Gynecology. Referral studies included the Alzheimer’s Disease Research Center (*n* = 73), Washington Heights Inwood Columbia Aging Project (*n* = 35), Testing Olfaction in Primary care to detect Alzheimer’s disease and other Dementias (*n* = 11), and Cognitive Reserve and Reference Ability Neural Network studies (*n* = 22), Imaging inflammation in elders with different clinical and biomarker profiles of Alzheimer’s disease (*n* = 2) Concerns About Memory Problems (*n* = 2). To be included in the current study, participants were required to have performed within normal limits on standard neuropsychological testing (demographically adjusted *z*-scores above −1.5) within the last 12 months (see Supplementary Table 1 for neuropsychological screening measures). Exclusion criteria included past or current history of neurological conditions such as aneurysm, stroke, traumatic brain injury, epilepsy, etc. This study was reviewed and approved by Columbia University’s Institutional Review Board (Protocol AAAR5197). Participants provided written informed consent.

### Subjective Cognitive Decline

Subjective cognitive decline was measured using a 20-item, age-anchored scale previously shown to detect a range of self-reported cognitive problems among cognitively normal older adults (see Chapman et al., 2021 for full description). In brief, the scale comprises 10 items assessing aspects of episodic memory, and 10 non-memory items covering aspects of attention, language, spatial function, and executive abilities. Participants are asked to judge the extent to which they have difficulty with each item as compared to others their age. Responses are given ordinally (0 = no problem – 6 = major problem) with a total score ranging from 0 to 120. Higher scores represent more subjective cognitive problems.

### Cognitive Markers of Subtle Cognitive Dysfunction

#### Short-Term Memory Binding

The short-term memory binding task (STMB) assesses the integration of multi-modal information in short-term memory (Parra et al., 2010, 2011). Specifically, this task assesses the ability to integrate two features of a stimulus (shape and color) and hold this representation in short-term memory (Parra et al., 2010). The STMB has been shown to be robust against age effects (Parra et al., 2009) and is specific to AD dementia (Della Sala et al., 2012) showing high sensitivity and specificity for pre-clinical AD (Parra et al., 2010). The main outcome of the STMB task represents total stimuli correctly recognized, ranging from 0 to 16 with higher scores indicating better performance (see Parra et al., 2009 for full description). To ensure the validity of the STMB outcome measure, participants are required to pass a practice trial in which they need to integrate shape and color with no demands on short-term memory. The ability to integrate these two features has been associated primarily with posterior parietal-occipital regions implicated in the ventral visual stream, regions hypothesized to be affected during the sub-hippocampal stages of AD, which suggests the task can detect the earliest stages of AD development (Parra et al., 2014).

#### The Loewenstein-Acevedo Scales of Semantic Interference and Learning

The Loewenstein-Acevedo Scales of Semantic Interference and Learning (LASSI-L) (Crocco et al., 2014) is a newly developed list-learning test that measures proactive semantic interference, retroactive interference, and the ability to recover from proactive semantic interference. Participants first read aloud a list of 15 words, List A, from three semantic categories: fruits, musical instruments, and articles of clothing. This is followed by a cued recall, with the three semantic categories as cues (“Can you tell me all the words on the list that were fruits?”). List A is then read again, followed by another cued recall. Then participants are presented with a new set of 15 words, List B, from the same semantic categories (fruits, musical instruments, and articles of clothing), followed by recall (*B1, susceptibility to proactive semantic interference*). The participants are presented with List B again, and recall (*B2, recovery from semantic interference*). Immediately following B2, participants are asked to recall all of the words from List A (*A3, susceptibility to retroactive semantic interference*). These three primary outcome measures (B1, B2, and A3) were included because they associate with biomarkers of AD such as amyloid load and volumetric loss. Specifically, this task has been shown to associate with amyloid accumulation in AD vulnerable regions such as the cingulate, precuneus, and frontal lobe in addition to volumetric and cortical reduction in the medial temporal lobe regions including the hippocampus (Loewenstein et al., 2016; Crocco et al., 2018).

### Metamemory

Metamemory was assessed with a modified feeling of knowing (FOK) (Cosentino et al., 2007). This task is comprised of four trials with five fictional trivia items per trial (e.g., Cole Porter attended law school in Chicago). Participants are instructed as follows: “During this task, I am going to tell you about five people. I will tell you their name and something about their background. Your task is to try to remember this information as best you can. Please listen carefully”). Following the first learning trial of the five fictional trivia, participants are queried regarding each of the five items, one at a time in a random order (e.g., Who attended law school in Chicago?). For each item, the examiner asks participants to estimate the likelihood of knowing the right answer (FOK judgment; “There are eight possible answers on the next page). Will you know which one is right (“Yes, Maybe, or No?”). After each individual FOK judgment, participants are asked to identify the correct answer (e.g., Porter) from eight possible choices including the correct answer as well as seven distractors. Item level judgments are given ordinal values of 0 (No), 0.5 (Maybe), and 1 (Yes). Memory for each item is scored as 0 (incorrect) and 1 (correct). There are four learning trials yielding a total of 20 FOK judgments. This task has been utilized in both patients with AD and healthy older adults (Cosentino et al., 2007, 2011a,2011b).

The primary metamemory outcome derived from this task is a resolution score representing a person’s ability to adjust judgments of performance in line with actual memory performance from one item to the next. This score is calculated *via* the Goodman Kruskal gamma statistic; a rank order correlation assessing the total number of concordances (C) across the test (instances in which judgments and performance both increase from one item to another) versus the total number of discordances (*D*; judgments for performance decrease when performance increases and vice versa). Gamma is calculated as (*C* − *D*)/(*C* + *D*). Following this formula, tests characterized by relatively more concordances than discordances will result in a gamma value closer to 1 (perfect resolution), while the opposite will result in a gamma value closer to −1. This calculation does not take into account the number of “ties” across items, that is, any two items in which either the judgment or memory values are equal. Therefore, if someone “ties” across all items (e.g., always judges that they will know the answer), gamma is not calculated (Cosentino et al., 2007).

### Statistical Analyses

All analyses were conducted with IBM SPSS v.26. Descriptive statistics were conducted for demographic, SCD, metamemory, and memory measures. Spearman one-tailed correlations were conducted to examine the bivariate associations between SCD, gamma and memory. To examine the moderating effect of metamemory on the association between SCD and memory outcomes, linear regression models were conducted in complete case data. Influential univariate outliers (standardized residuals >3 or <−3) and multivariate outliers (determined through Mahalanobis distance) were examined for each model. To test for a specification error in the moderation models, namely that there is curvilinearity in the relation of each predictor to the dependent variable, quadratic effects of both SCD and gamma were included in separate models (Lubinski and Humphreys, 1990). Next, models were rerun without cases of gamma = 1 to examine if the frequency of these cases biased results. Finally, sensitivity analyses were conducted with imputed case data. A regression based multiple imputation approach was utilized for imputation. The pooled data from 25 imputations were utilized to obtain the estimates of variables in the model. All models were adjusted for demographic factors including age, self-reported gender, race, and education. In addition, a False Discovery Rate correction was implemented to complete cases that adjusted for the main comparisons of interest in the study which included demographical associations with main variables of interest, main effects of SCD and gamma on cognitive outcomes as well as their interactive effects.

## Results

### Descriptives

Table 1 summarizes descriptives of demographics, cognitive, and metacognitive measures in the sample. All participants completed the SCD questionnaire (*n* = 157). A total of 156 participants completed the metamemory test, and 1 refused. Of the 156, 29 participants had ties across their pairs in the metamemory test and therefore gamma could not be calculated. The LASSI-L was available for 98 participants, as it was added to the study battery later. Finally, 9 participants failed to pass the validity trial for the STMB and one refused to complete due to color blindness leaving a total sample of 79 participants with all available measures. Descriptives are thus provided for these 79 participants with available data across all measures in Table 1. Demographics were found to be associated with gamma and cognitive outcomes. Specifically, age was negatively associated with gamma, susceptibility and ability to recover from proactive interference and retroactive interference (*r* range = −0.20, −0.29, *p* range = 0.004, 0.042). Greater levels of educational attainment were significantly associated with better performance in trials assessing susceptibility and ability to recover from proactive interference as well as retroactive interference (*r* range = 0.21, 0.36, *p* range = <0.001, 0.035). With regards to race, significant differences were observed with regards to performance in the STMB task only wherein White participants had higher performance (*M* = 10.61, *SD* = 9.56) than Black participants (*M* = 9.56, *SD* = 2.27) [*t*(77) = 2.24, *p* = 0.028], however, this difference did not withstand adjustment for educational attainment. No differences were observed in SCD, gamma nor cognitive outcomes regarding gender.

**TABLE 1:** Demographics, subjective cognitive decline (SCD), memory and metamemory (*n* = 79).

	*M* (*SD*) or *n* (%)	Sample range
Age (years)	74.4 (6.1)	62 – 88
Education (years)	15.9 (2.5)	10 – 20
Gender – female participants	61 (77%)	
Race		
Black participants	25 (32%)	–
White participants	54 (68%)	–
SCD (0 – 120)	22.2 (16.9)	0 – 60
Metamemory – gamma (−1 – 1)	0.6 (0.5)	−1 – 1
LASSI-L outcomes		
LASSI-L B1 (0 – 15)	8.3 (3.0)	1 – 15
LASSI-L B2 (0 – 15)	11.9 (2.6)	6 – 15
LASSI-L A3 (0 – 15)	9.7 (2.5)	4 – 15
STMB	10.2 (2.0)	5 – 14

### Bivariate Analyses

Table 2 summarizes bivariate association between SCD, metamemory and cognitive outcomes. Increased SCD was associated with worse recall on B1 and A3 indicating that individuals endorsing more complaints had increased susceptibility to semantic proactive and retroactive interference. For sensitivity analyses with imputed data please see Supplementary Table 2.

**TABLE 2:** Bivariate associations between SCD, cognition and metamemory (*n* = 79).

	SCD			Metamemory – gamma		
	*r*	*p*	CI	*r*	*p*	CI
Metamemory – gamma	−0.05	0.32	−0.26, 0.18	–	–	–
LASSI-L outcomes						
LASSI-L B1	−**0.30**	**0.003**	−**0.51,**−**0.08**	–0.01	0.470	−0.25, 0.26
LASSI-L B2	−0.07	0.270	−0.31, 0.15	0.012	0.457	−0.19, 0.24
LASSI-L A3	−**0.19**	**0.047**	−**0.42, 0.03**	–0.01	0.457	−0.26, 0.21
STMB	−0.15	0.099	−0.36, 0.10	–0.015	0.446	−0.19, 0.20

*Confidence intervals (CI) calculated from 1,000 bootstrapping samples. Significant associations bolded.*

### Regression Models

Table 3 summarizes main effect models without interaction terms and Table 4 summarizes results of the interactive effect of metamemory (gamma) with SCD on cognitive outcomes. Increased age, SCD, being male and having lower educational attainment was associated with increased susceptibility to proactive semantic interference reflected by lower recall on B1. In the second main effect model with B2 as the outcome, increased age was associated with reduced ability to recover from proactive interference. In the third main effect model examining A3 as an outcome, increased age was associated with increased susceptibility to retroactive semantic interference. Finally, in the main effect model of STMB, there were no variables that individually predicted STMB. With regard to moderation models, a significant interaction effect of metamemory and SCD was observed for B1 (*susceptibility to proactive semantic interference*) such that individuals with higher levels of metamemory had a stronger negative association between SCD and proactive interference. Metamemory’s also moderated the association SCD and B2 (*ability to recover from proactive semantic interference*).

**TABLE 3:** Main effect models of SCD, gamma and demographic associations with LASSI-L and STMB outcomes.

	*B* (*SE*)	Std. *B*	*p*-value
**SCD = >B1**			
SCD	−0.41 (0.14)	–0.30	0.003
Gamma	−0.28 (0.54)	–0.05	0.613
Age	−0.14 (0.05)	–0.29	0.004
Gender (0 = men, 1 = women)	1.60 (0.68)	0.23	0.020
Education	0.35 (0.13)	0.29	0.008
Race (0 = white, 1 = black)	−0.52 (0.68)	–0.08	0.450
**SCD = >B2**			
SCD	−0.09 (0.13)	–0.07	0.514
Gamma	−0.14 (0.53)	–0.03	0.798
Age	−0.12 (0.05)	–0.30	0.009
Gender (0 = men, 1 = women)	1.08 (0.65)	0.18	0.101
Education	0.17 (0.13)	0.17	0.174
Race (0 = white, 1 = black)	−0.29 (0.66)	–0.05	0.660
**SCD = > A3**			
SCD	−0.22 (0.12)	–0.19	0.080
Gamma	−0.25 (0.49)	–0.06	0.606
Age	−0.11 (0.04)	–0.27	0.015
Gender (0 = men, 1 = women)	0.85 (0.61)	0.15	0.163
Education	0.18 (0.12)	0.19	0.125
Race (0 = white, 1 = black)	−0.36 (0.61)	–0.07	0.555
**SCD = > STMB**			
SCD	−0.14 (0.10)	–0.15	0.172
Gamma	−0.20 (0.41)	–0.05	0.636
Age	−0.59 (0.04)	–0.18	0.111
Gender (0 = men, 1 = women)	0.39 (0.51)	0.08	0.451
Education	0.14 (0.10)	0.18	0.148
Race (0 = white, 1 = black)	−0.74 (0.52)	–0.18	0.155

**TABLE 4:** Moderation models of gamma on SCD’s associations with cognitive outcomes.

	*B* (*SE*)	Std. *B*	*p*-value
**SCD = >B1**			
SCD	0.07 (0.21)	0.05	0.745
Gamma	2.90 (1.22)	0.53	0.020
SCD* gamma	**−0.72 (0.25)**	**−0.71**	**0.005**
Age	−0.13 (0.05)	–0.26	0.006
Gender (0 = men, 1 = women)	1.50 (0.65)	0.21	0.023
Education	0.33 (0.12)	0.28	0.009
Race (0 = white, 1 = black)	−0.64 (0.65)	–0.10	0.328
**SCD = >B2**			
SCD	0.27 (0.21)	0.228	0.197
Gamma	2.23 (1.21)	0.474	0.069
SCD* gamma^	**−0.54 (0.25)**	**−0.62**	**0.034**
Age	−0.12 (0.05)	–0.28	0.013
Gender (0 = men, 1 = women)	1.01(0.637)	0.17	0.117
Education	0.16 (0.122)	0.16	0.197
Race (0 = white, 1 = black)	−0.3 (0.642)	–0.07	0.555
**SCD = >A3**			
SCD	0.02 (0.20)	0.02	0.924
Gamma	1.30 (1.14)	0.29	0.259
SCD* gamma	−0.35 (0.24)	–0.43	0.138
Age	−0.10 (0.04)	–0.26	0.020
Gender (0 = men, 1 = women)	0.81 (0.60)	0.14	0.185
Education	0.17 (0.12)	0.18	0.140
Race (0 = white, 1 = black)	−0.42 (0.61)	–0.08	0.489
**SCD = >STMB**			
SCD	−0.07 (0.17)	–0.08	0.682
Gamma	0.28 (0.98)	0.08	0.776
SCD* gamma	−0.11 (0.20)	–0.16	0.593
Age	−0.06 (0.04)	–0.17	0.126
Gender (0 = men, 1 = women)	0.37 (0.52)	0.08	0.471
Education	0.14 (0.10)	0.18	0.157
Race (0 = white, 1 = black)	−0.76 (0.52)	–0.18	0.148

Significant interaction terms bolded. ^Did not survive FDR correction. *Represents the interaction terms where SCD is multiplied by metamemory.

One multivariate outlier was found in the moderation models with B1 and B2 as outcomes; exclusion of this outlier did not change results. In order to examine the influence of gamma = 1, moderation regression models were rerun without these cases (*n* = 60); the significant moderation effect remained. Specifically, the moderating effect of gamma was significant in models with B1 and B2 as outcomes (*p* = 0.006; *p* = 0.020). Third, in order to examine specification error, moderation models were rerun with quadratic terms of SCD and gamma. The moderation effect of gamma remained significant (*p* = 0.009) for the model with B1 as an outcome but not B2 where the effect lost significance at the margin (*p* = 0.055). Further, given that various measures had missing data, sensitivity analyses were conducted with all imputed data. Please see Supplementary Tables 3, 4. Whilst most results remained consistent, the moderating effect of gamma for models with B2 as an outcome lost significance (*p* = 0.085) consistent with our FDR correction.

## Discussion

This study examined the extent to which metamemory moderated the association between SCD and memory abilities in older adults. Consistent with previous work showing an association between SCD and rigorous measures of subtle cognitive dysfunction (Chapman et al., 2021), bivariate associations revealed that individuals with higher SCD had weaker performance on select list learning measures including greater susceptibility to both proactive interference and retroactive interference. With regard to the moderating role of metamemory, results from this study support the idea that in general, SCD is more strongly linked to memory abilities among individuals with better metamemory. Indeed, metamemory moderated the association between SCD and susceptibility to proactive interference Metamemory did not, however, moderate the association between SCD and retroactive interference or short-term memory binding. Below we offer potential interpretations for these findings and discuss current issues in the measurement and conceptualization of SCD more broadly, beginning with the variable associations between SCD and the memory outcomes selected for the current study.

The selective associations between SCD and only two of four memory outcomes, all previously shown to be sensitive to preclinical AD (Parra et al., 2010; Loewenstein et al., 2016; Crocco et al., 2018), was somewhat unexpected. For example, both proactive and retroactive interference on the LASSI-L have been linked to total cortical loading of amyloid and the precuneus specifically, among cognitively normal older adults (Loewenstein et al., 2016). In fact, the ability to *recover* from proactive interference has repeatedly been shown to be more sensitive to pre-clinical AD than other LASSI markers (Loewenstein et al., 2016, 2017). It is thus not immediately clear why SCD relates differently to each of these metrics. Susceptibility to proactive interference, associated with SCD in the current study, is assessed by measuring recall of List B after two study trials of List A. Recovery from proactive interference, not currently associated with SCD, is defined as recall of List B after its second presentation. It may be that in the current cognitively normal sample, there is little variability in performance after studying this list twice, limiting the degree to which it maps onto SCD. Indeed, average scores were higher (11.9) and the minimum score higher (6) than on the susceptibility metric (8.3 and 1, respectively). Nevertheless, the selective associations between SCD and increased susceptibility to proactive and retroactive interference may reflect specific early dysfunctions in cognitive control mechanisms. Previous research has shown that individuals with reduced working memory capacity (Rosen and Engle, 1998; Brewin and Smart, 2005) or inhibitory control (Anderson et al., 2000; Anderson, 2003; Anderson and Levy, 2007) tend to be more susceptible to interference effects and intrusive thoughts. Subtle changes in these cognitive control mechanisms could impact the use of specific and more effective retrieval mechanisms (Anderson and Levy, 2007; Unsworth, 2016, 2019).

Unexpectedly, SCD was also unrelated to short-term memory binding, the latter measure having previously been associated with SCD in a subset of this same cohort (Chapman et al., 2021). It is important to keep in mind, however, that while both the LASSI-L and STMB are sensitive to preclinical AD, their neural underpinnings are not synonymous. As highlighted earlier, LASSI-L measures have been associated with amyloid load in key AD regions such as cingulate, precuneus, frontal lobe as well as volumetric and cortical integrity of medial temporal lobe regions including the hippocampus. In contrast, the STMB has been associated primarily with posterior parietal-occipital regions implicated in the ventral visual stream, regions hypothesized to be affected during the sub-hippocampal stages of AD (Parra et al., 2014). As such, depending on the regional distribution of potential brain changes among individuals in a given sample, the extent to which SCD maps onto one or another cognitive measure will likely differ.

The inconsistency of metamemory as a moderator was also unexpected. While the size and direction of the moderation effect were generally comparable across different outcome measures, the moderating effect was only significant for SCD and measures of proactive interference (susceptibility to and recovery from), but not retroactive interference or short-term memory binding. There are several factors that could have led to this discrepancy. First, the link between SCD and memory itself is variable as discussed above. It may not be feasible to detect a significant moderation effect in situations where SCD is not even weakly associated with a specific memory outcome, as was the case for STMB in the current study. A second potential issue is that metamemory itself is heterogeneous, consisting of two broad categories: monitoring (i.e., what you know about your memory) and control (i.e., how you manage your memory). Monitoring, the focus of the current study, is itself multi-dimensional and can be operationalized in a number of ways that capture individuals’ confidence level (i.e., calibration) as well as their ability to adjust their expectations for performance as it varies over the course of a test (i.e., resolution). Furthermore, metamemory can be measured at different levels including an item-by-item basis (e.g., *will you know the answer to this question?*), or a summary level (e.g., *how many answers will you know overall?*) as well as at different points in time, including prior to or following memory performance (Nelson, 1984, 1990). Different studies have revealed nuances in the correlates of individual metamemory measures depending on a variety of factors including the score that is used (calibration versus resolution), the level at which it is measured (item versus summary), and the population in which it is measured (cognitively normal older adults versus AD) (Kikyo et al., 2002; Maril et al., 2003; Kikyo and Miyashita, 2004; Chua et al., 2006, 2009; Cosentino et al., 2007; Bertrand et al., 2018). From a cognitive perspective, aging studies have shown that confidence in retrieval judgments may be susceptible to variations in memory functioning (Hertzog et al., 2010, 2021). In line with this, reduced memory abilities in older adults may limit their access to diagnostic cues necessary to make accurate metacognitive judgments (Dunlosky and Metcalfe, 2008). Alternatively, older adults might have access to adequate cues but be unable to make valid inferences to reach accurate metacognitive judgments, possibly due to age-related changes in pre-frontal networks (Perrotin et al., 2008; Thomas et al., 2011; Fleming and Dolan, 2012). Given the seeming susceptibility in the current cohort to interference effects, and the moderating effects in this domain, we could also speculate that early vulnerability in frontal medial regions results in compromise to inferential judgments and resultingly to less accurate metacognitive judgments. Additional work is needed to tease apart the underlying cognitive as well as neuroanatomical substrates of both the susceptibility to interference and the moderating effects of metamemory ability.

In conclusion, results partially support our hypothesis that metamemory would moderate the association between SCD and memory performance, and provide rationale for consideration of metamemory when evaluating the accuracy of SCD. However, this study was not without limitations. First, the current sample included only participants with all available measures which reduced the sample significantly. However, in order to address this limitation, a multiple imputation approach was conducted in sensitivity analyses which revealed no significant differences between the initial model and the imputed model with the exception of the interactive effect of gamma and SCD on B2, also indicated in the False Discovery Rate adjusted *p*-values applied to complete-case analyses. A second limitation was that in 24 participants, gamma was not computed due to ties (i.e., no variability in either their FOK judgments or performance accuracy, with the majority of these cases always indicating “yes” for the FOK judgment with accuracy scores = 1). These cases *could* be considered as having perfect metamemory, highlighting a possible limitation of our task which for some participants may have a ceiling effect. A greater number of items within each learning trial would increase the likelihood of calculable gamma scores and provide a more comprehensive measure of metamemory in older adults. Another possible limitation was the relatively low level of SCD reported within this sample, along with possible ceiling effects on some cognitive measures which also may have reduced the strength of associations between SCD and cognition, as well as the moderating role of metamemory. Finally, the cohort included in this sample primarily included individuals drawn from other ongoing research studies rather than individuals presenting to a memory disorders clinic, which could skew not only the distribution of SCD but the level of concern regarding SCD, a factor known to increase SCD’s utility as a maker of preclinical AD (Jessen et al., 2010). Ideally, this study would have included sensitivity analyses to explore the effects of community/research recruited versus clinically recruited. This analysis, however, was not possible given that only 13/157 individuals were clinic recruited. There are numerous ways in which we are currently tailoring our ongoing study of SCD, including increasing SCD screenings and referrals from the community and local clinical practices to enroll individuals with higher levels of SCD. Moreover, we are tracking participants longitudinally to examine the extent to which SCD predicts decline over time, as well as the extent to which change in SCD is more predictive than a single SCD assessment. The current literature is mixed; For example, while Drouin et al. (2021) found that subjective memory change predicted longitudinal memory change, Hertzog et al. (2018) found that subjective memory change was more related to current memory complaint rather than an indicator of actual memory change.

This study also had a number of considerable strengths including the prospective, rigorous assessment of SCD using an age-anchored framework shown to relate more closely than other measurement frameworks (e.g., comparing one’s memory to 5 years ago) to objective measures of cognition (Perrotin et al., 2012; Tandetnik et al., 2015; Chapman et al., 2021). Another notable strength was the inclusion of objective metamemory testing, as well as two novel memory tests sensitive to pre-clinical AD, all of which have rarely if ever been combined in a single cohort. Finally, all participants completed comprehensive neuropsychological testing to ensure that they did not meet criteria for Mild Cognitive Impairment. Ongoing work, in addition to enriching our sample with individuals who present to the clinic with complaints, is examining not only the relative contribution of metamemory as a moderator, but of other person factors such as mood, personality, and attitudes about aging (Chapman et al., in preperation). Together, these analyses will continue to inform the way in which SCD can be optimized as a marker of pre-clinical AD.

## Data Availability Statement

The datasets presented in this article are not readily available because due to IRB restrictions we cannot share the data. Requests to access the datasets should be directed to StC, sc2460@cumc.columbia.edu.

## Ethics Statement

The studies involving human participants were reviewed and approved by IRB COLUMBIA UNIVERSITY. The patients/participants provided their written informed consent to participate in this study.

## Author Contributions

All authors listed have made a substantial, direct, and intellectual contribution to the work, and approved it for publication.

## Conflict of Interest

The authors declare that the research was conducted in the absence of any commercial or financial relationships that could be construed as a potential conflict of interest.

## Publisher’s Note

All claims expressed in this article are solely those of the authors and do not necessarily represent those of their affiliated organizations, or those of the publisher, the editors and the reviewers. Any product that may be evaluated in this article, or claim that may be made by its manufacturer, is not guaranteed or endorsed by the publisher.
