# Supplementary material for: Subjective Cognitive Decline Is More Accurate When Metamemory Is Better
Source: Front Aging Neurosci. 2022 Mar 9;14:787552. doi: 10.3389/fnagi.2022.787552 (PMC8965471; doi:10.3389/fnagi.2022.787552)

**APPENDIX**

| Supplementary Table S1. Cognitive assessments used as screening for enrollment in the study |
| --- |
| *Cognitive Screening Tests for inclusion in study* |
| **Memory** |
| Selective Reminding Test Total Learning |
| Selective Reminding Test Delayed Recall |
| Hopkins Verbal Learning Test Total Learning |
| Hopkins Verbal Learning Test Delayed Recall |
| **Executive Function** |
| Trail Making Test B Total Completion Time |
| Color Trails Test B Total Completion Time |
| **Language** |
| Semantic Fluency (Animals) Total number |
| Phonemic Fluency (FAS or CFL) Total number |
|  |

(Brandt, 1991; Buschke, 1984; D'Elia, Satz, Uchiyama, & White, 1996; Lezak, 1995; Reitan & Wolfson, 1985)

Supp. References

Buschke, H., Cued recall in Amnesia. Journal of Clinical Neuropsychology, 1984. 6(4): p. 433-440.

Brandt, J., The hopkins verbal learning test: Development of a new memory test with six equivalent forms. Clinical Neuropsychologist, 1991. 5(2): p. 125-142.

Reitan, R.M. and D. Wolfson, The Halstead–Reitan Neuropsycholgical Test Battery: Therapy and clinical interpretation. 1985, Tucson, AZ: Neuropsychological Press.

D'Elia, L., et al., Color trails test. 1996: PAR Odessa, FL.

Lezak, M.D., Neuropsychological assessment, 3rd ed. Neuropsychological assessment, 3rd ed. 1995, New York, NY, US: Oxford University Press. xviii, 1026-xviii, 1026.

Table S2. Bivariate associations between SCD, cognition and metamemory with imputed data (n=157)

|  | SCD | | Metamemory - Gamma | |
| --- | --- | --- | --- | --- |
|  | *r* | *p* | *r* | *p* |
| Metamemory – Gamma | -.07 | .218 | - | - |
| LASSI-L outcomes |  |  |  |  |
| LASSI-L B1 | **-.30** | **<.001** | .03 | .376 |
| LASSI-L B2 | -.12 | .104 | .04 | .353 |
| LASSI-L A3 | **-.17** | **.036** | .02 | .431 |
| STMB | -.07 | .207 | -.03 | .392 |

**Note.** Significant results bolded

Table S3. Main effect models of SCD, gamma and demographic associations with LASSI-L and STMB outcomes with imputed data (n=157)

| *SCD* => *B1* | B | SE | p-value |
| --- | --- | --- | --- |
| SCD | **-0.34** | **0.12** | **0.004** |
| Gamma | -0.07 | 0.45 | 0.878 |
| Age | -0.10 | 0.04 | 0.011 |
| Gender  0, Men relative to 1, women | -0.89 | 0.54 | 0.099 |
| Education | **0.26** | **0.10** | **0.013** |
| Race |  |  |  |
| 0, White relative to 2, Black race | 0.61 | 0.61 | 0.316 |
| 1, Other relative to 2, Black race | -0.04 | 1.16 | 0.972 |
| *SCD* => *B2* |  |  |  |
| SCD | -0.09 | 0.10 | 0.373 |
| Gamma | 0.02 | 0.39 | 0.965 |
| Age | **-0.08** | **0.03** | **0.011** |
| Gender  0, Men relative to 1, women | -0.74 | 0.44 | 0.093 |
| Education | 0.14 | 0.09 | .113 |
| Race |  |  |  |
| 0, White relative to 2, Black race | 0.38 | 0.50 | 0.447 |
| 1, Other relative to 2, Black race | 0.29 | 1.05 | 0.786 |
| *SCD* => *A3* |  |  |  |
| SCD | -0.16 | 0.10 | .108 |
| Gamma | -0.14 | 0.38 | .708 |
| Age | -0.09 | 0.03 | **.006** |
| Gender  0, Men relative to 1, women | -0.71 | 0.46 | .130 |
| Education | 0.17 | 0.09 | .068 |
| Race |  |  |  |
| 0, White relative to 2, Black race | 0.46 | 0.50 | .364 |
| 1, Other relative to 2, Black race | -1.24 | 1.05 | .240 |
| *SCD* => *STMB* |  |  |  |
| SCD | -0.06 | 0.09 | 0.468 |
| Gamma | -0.20 | 0.36 | 0.576 |
| Age | -0.05 | 0.02 | 0.059 |
| Gender  0, Men relative to 1, women | -0.27 | 0.38 | 0.484 |
| Education | 0.06 | 0.08 | 0.498 |
| Race |  |  |  |
| 0, White relative to 2, Black race | 0.52 | 0.44 | 0.237 |
| 1, Other relative to 2, Black race | -0.50 | 0.93 | 0.594 |

**Note**. Significant effects bolded

Table S5. Moderation models of gamma on SCD’s associations with cognitive outcomes with imputed data (n=157)

| *SCD* => *B1* | B | SE | p-value |
| --- | --- | --- | --- |
| SCD | -0.09 | 0.17 | 0.582 |
| Gamma | 1.64 | 0.95 | 0.085 |
| **SCD* Gamma** | **-0.4** | **0.20** | **0.047** |
| Age | **-0.09** | **0.04** | **0.014** |
| Gender  0, Men relative to 1, women | -0.90 | 0.53 | 0.092 |
| Education | **0.24** | **0.10** | **0.20** |
| Race |  |  |  |
| 0, White relative to 2, Black race | 0.67 | 0.61 | .274 |
| 1, Other relative to 2, Black race | -0.11 | 1.14 | .923 |
|  |  |  |  |
| *SCD* => *B2* |  |  |  |
| SCD | 0.09 | 0.14 | .524 |
| Gamma | 1.31 | 0.84 | .121 |
| **SCD* Gamma** | -0.31 | 0.18 | .085 |
| Age | **-0.08** | **0.03** | **.013** |
| Gender  0, Men relative to 1, women | 0.09 | 0.14 | .086 |
| Education | 0.12 | 0.09 | .171 |
| Race |  |  |  |
| 0, White relative to 2, Black race | 0.44 | 0.50 | .377 |
| 1, Other relative to 2, Black race | 0.16 | 1.04 | .874 |
| *SCD* => *A3* |  |  |  |
| SCD | -0.01 | 0.14 | .959 |
| Gamma | 0.92 | 0.8 | .252 |
| SCD* Gamma | -0.25 | 0.18 | .151 |
| Age | -0.09 | 0.03 | .007 |
| Gender  0, Men relative to 1, women | -0.72 | 0.46 | .122 |
| Education | 0.15 |  | .098 |
| Race |  |  |  |
| 0, White relative to 2, Black race | 0.51 | 0.50 | .316 |
| 1, Other relative to 2, Black race | -1.34 | 1.05 | .201 |
| *SCD* => *STMB* |  |  |  |
| SCD | -0.05 | 0.12 | .698 |
| Gamma | -0.09 | 0.72 | .899 |
| SCD* Gamma | -0.03 | 0.16 | .869 |
| Age | -0.05 | 0.02 | .060 |
| Gender  0, Men relative to 1, women | -0.27 | 0.38 | .483 |
| Education | 0.05 | 0.02 | .516 |
| Race |  |  |  |
| 0, White relative to 2, Black race | 0.52 | 0.44 | .233 |
| 1, Other relative to 2, Black race | -0.51 | 0.92 | .584 |

Note. Significant terms bolded.

Supplementary Figure 1. Distribution of LASSI-L B1 cued recall.


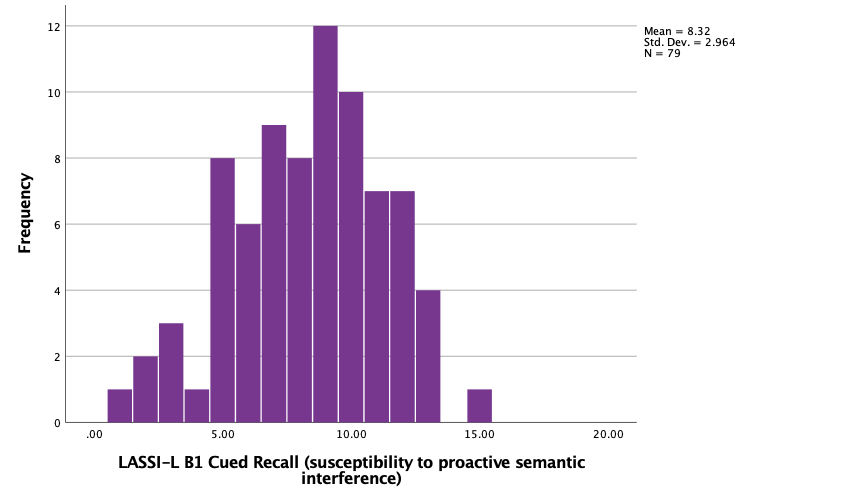


Supplementary Figure 2. Distribution of LASSI-L B2 cued recall


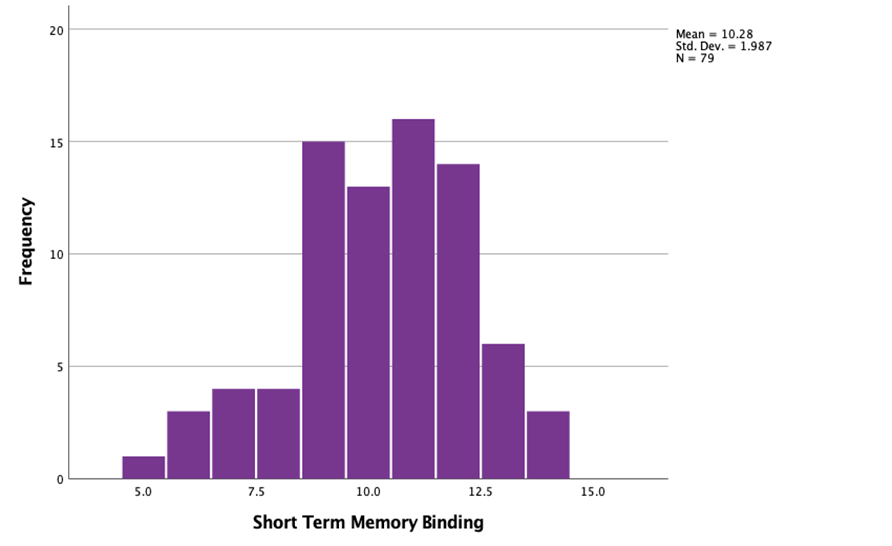


Supplementary Figure 3. Distribution of LASSI-L A3 cued recall


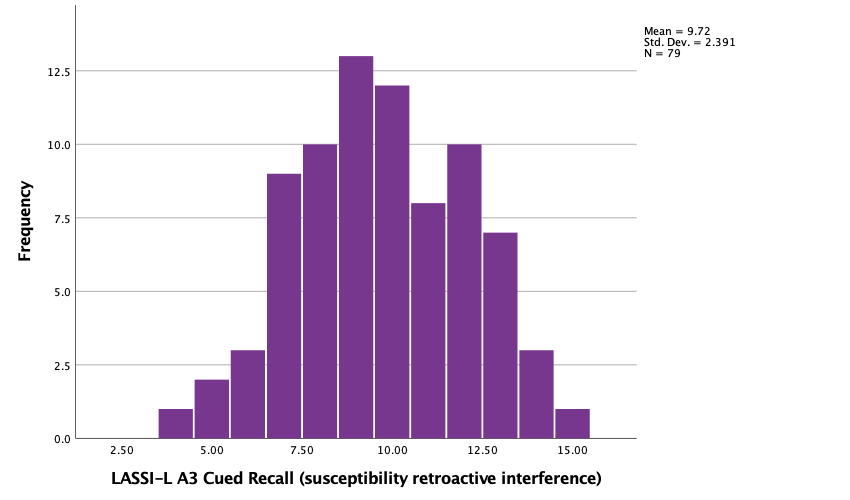


Supplementary Figure 4. Distribution of the Short Term Memory Binding Test.


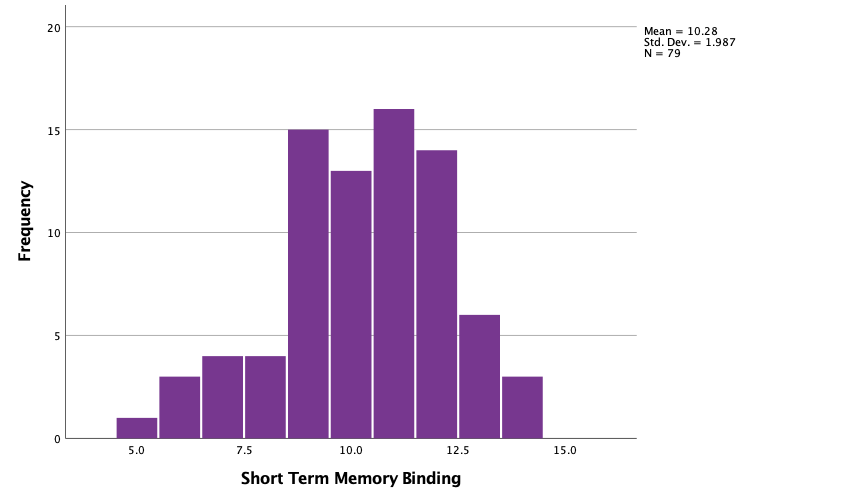

Supplement: Supplementary file 1 [file Table_1.docx]
